# Drug-induced notched T waves

**DOI:** 10.1007/s12471-021-01544-z

**Published:** 2021-02-09

**Authors:** T. A. C. de Vries, J. Seelig, R. Pisters, M. E. W. Hemels

**Affiliations:** 1 Department of Cardiology, Rijnstate Hospital, Arnhem, The Netherlands; 2 Department of Cardiology, Amsterdam UMC, University of Amsterdam, Amsterdam Cardiovascular Sciences, Amsterdam, The Netherlands; 3 Cardiovascular Research Institute Maastricht (CARIM), Maastricht, The Netherlands; 4 Department of Cardiology, Radboud University Medical Centre, Nijmegen, The Netherlands

**Fig. 1:**
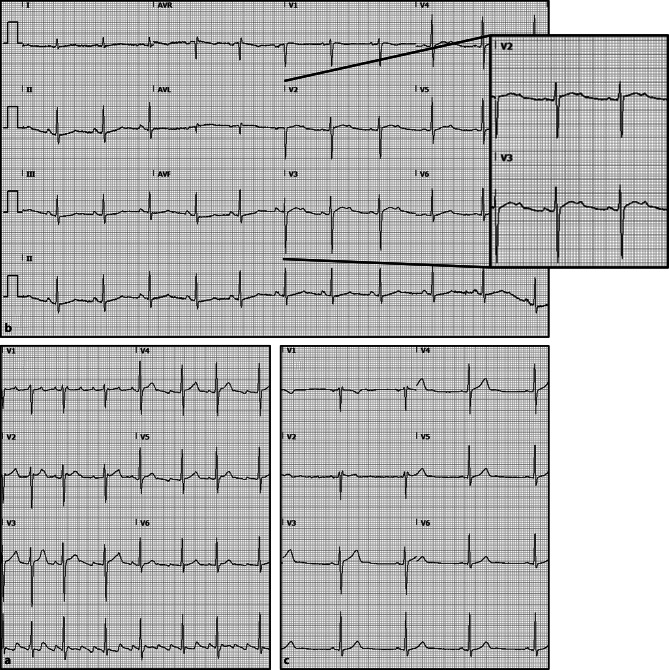
**a** Electrocardiogram recorded before administration of ibutilide. A typical, counter-clockwise, atrial flutter with an alternating atrioventricular conduction block. **b** Electrocardiogram recorded 5 min after intravenous administration of ibutilide. Besides successful conversion to sinus rhythm, QTc prolongation (460 ms) can be observed along with a positive deflection in the downward slope of the T waves in leads V2–V5. **c** Electrocardiogram 2 weeks after admittance to the emergency department. The QTc interval has returned to normal (390 ms)

A 27-year-old man was admitted with a recurrence of atrial flutter. He had no other relevant medical history and had used 80 mg sotalol as a ‘pill-in-the-pocket’ in addition to sotalol 80 mg twice daily. His current (Fig. 1a) and previous electrocardiograms showed a normal QTc interval. Serum potassium levels were within the normal range. One milligram of ibutilide was administered intravenously, preceded by 750 mg of intravenous magnesium as a precautionary measure.

An electrocardiogram recorded 5 min after ibutilide administration showed, aside from conversion to sinus rhythm, a prolonged QTc of 460 ms (from 390 ms) and notched T waves in leads V2–V5 (Fig. 1b). Notched T waves are classically described in congenital long QT syndrome (LQTS) [1], especially in LQTS type 2 where these T waves are reported in 63% of cases [2]. LQTS type 2 is caused by loss of function of the *KCNH2* (*hERG*) gene, which codes for the rapid delayed rectifier potassium channel (*I*_Kr_) and plays an important role in the repolarisation of the myocardial cell. However, drugs not genetics—in particular class III antiarrhythmic drugs including sotalol and ibutilide—are the most common cause of interference with *I*_Kr_. Although less frequently documented, such drugs can also cause notched T waves [3, 4].

This case underlines the risks involved when multiple QTc-prolonging drugs are administered, even in those without other risk factors for QTc prolongation [5]. Granting that the usefulness of intravenous magnesium prior to ibutilide is uncertain, it is a simple strategy that may potentially prevent torsade de pointes. Our patient was discharged after 4 h of continuous monitoring during which no arrhythmias occurred. An electrocardiogram recorded 2 weeks after admittance confirmed the QTc interval had returned to normal (Fig. 1c).
